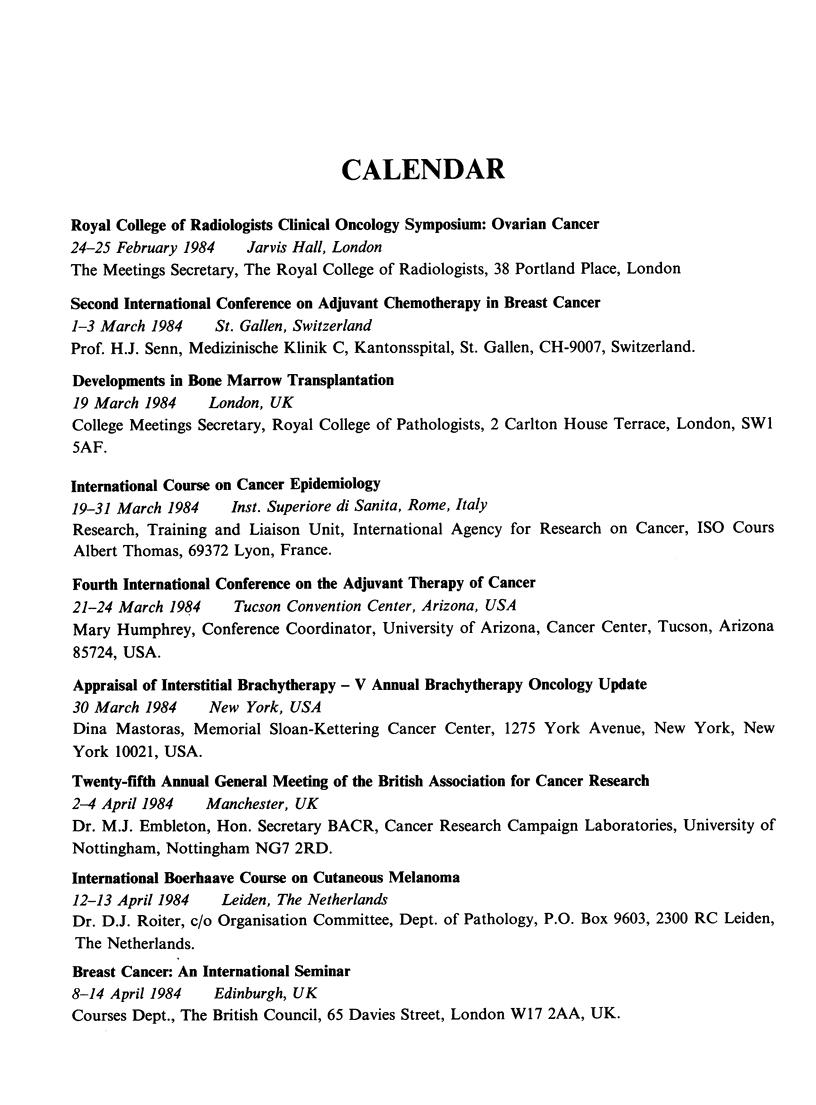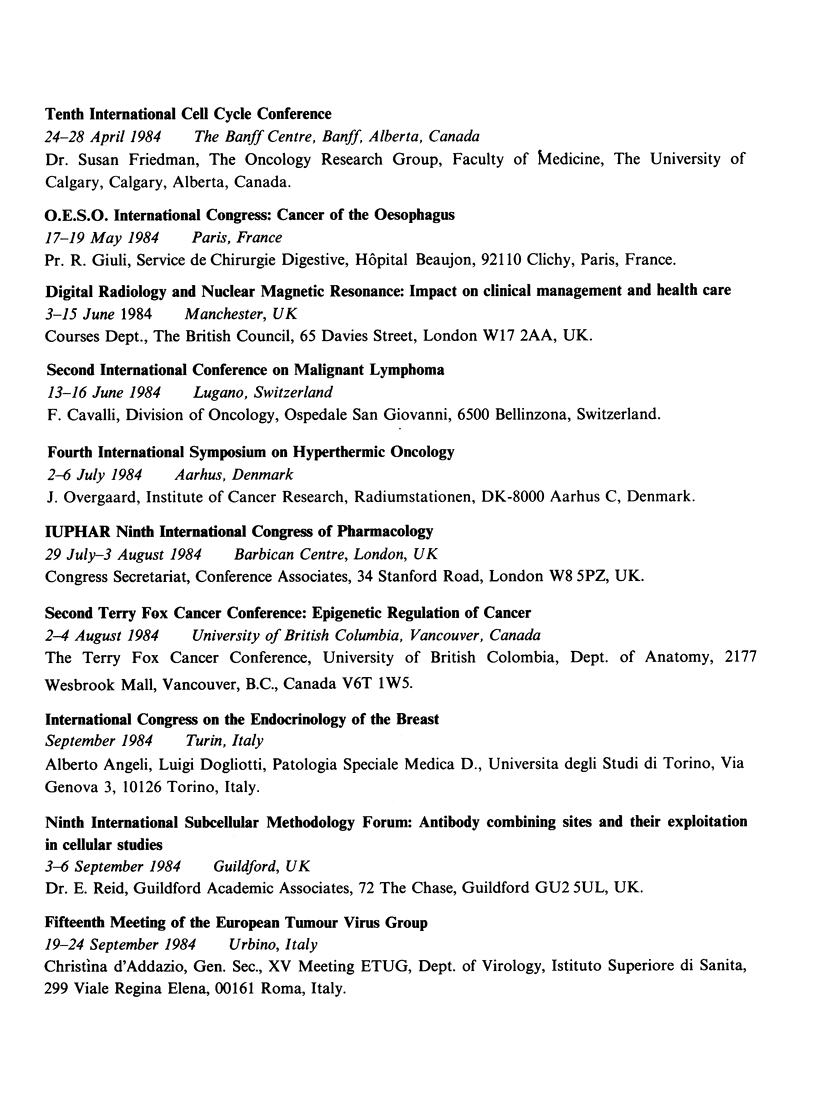# Calendar

**Published:** 1984-02

**Authors:** 


### CALENDAR

**Royal College of Radiologists Clinical Oncology Symposium: Ovarian Cancer**
*24–25 February 1984* *Jarvis Hall, London*
The Meetings Secretary, The Royal College of Radiologists, 38 Portland Place, London

**Second International Conference on Adjuvant Chemotherapy in Breast Cancer**
*1–3 March 1984* *St. Gallen, Switzerland*
Prof. H.J. Senn, Medizinische Klinik C, Kantonsspital, St. Gallen, CH-9007, Switzerland.

**Developments in Bone Marrow Transplantation**
*19 March 1984* *London, UK*
College Meetings Secretary, Royal College of Pathologists, 2 Carlton House Terrace, London, SW1 5AF.

**International Course on Cancer Epidemiology**
*19–31 March 1984* *Inst. Superiore di Sanita, Rome, Italy*
Research, Training and Liaison Unit, International Agency for Research on Cancer, ISO Cours Albert Thomas, 69372 Lyon, France.

**Fourth International Conference on the Adjuvant Therapy of Cancer**
*21–24 March 1984* *Tucson Convention Center, Arizona, USA*
Mary Humphrey, Conference Coordinator, University of Arizona, Cancer Center, Tucson, Arizona 85724, USA.

**Appraisal of Interstitial Brachytherapy – V Annual Brachytherapy Oncology Update**
*30 March 1984* *New York, USA*
Dina Mastoras, Memorial Sloan-Kettering Cancer Center, 1275 York Avenue, New York, New York 10021, USA.

**Twenty-fifth Annual General Meeting of the British Association for Cancer Research**
*2–4 April 1984* *Manchester, UK*
Dr. M.J. Embleton, Hon. Secretary BACR, Cancer Research Campaign Laboratories, University of Nottingham, Nottingham NG7 2RD.

**International Boerhaave Course on Cutaneous Melanoma**
*12–13 April 1984* *Leiden, The Netherlands*
Dr. D.J. Roiter, c/o Organisation Committee, Dept. of Pathology, P.O. Box 9603, 2300 RC Leiden, The Netherlands.

**Breast Cancer: An International Seminar**
*8–14 April 1984* *Edinburgh, UK*
Courses Dept., The British Council, 65 Davies Street, London W17 2AA, UK.

**Tenth International Cell Cycle Conference**
*24–28 April 1984* *The Banff Centre, Banff, Alberta, Canada*
Dr. Susan Friedman, The Oncology Research Group, Faculty of Medicine, The University of Calgary, Calgary, Alberta, Canada.

**O.E.S.O. International Congress: Cancer of the Oesophagus**
*17–19 May 1984* *Paris, France*
Pr. R. Giuli, Service de Chirurgie Digestive, Hôpital Beaujon, 92110 Clichy, Paris, France.

**Digital Radiology and Nuclear Magnetic Resonance: Impact on clinical management and health care**
*3–15 June* 1984 *Manchester, UK*
Courses Dept., The British Council, 65 Davies Street, London W17 2AA, UK.

**Second International Conference on Malignant Lymphoma**
*13–16 June 1984* *Lugano, Switzerland*
F. Cavalli, Division of Oncology, Ospedale San Giovanni, 6500 Bellinzona, Switzerland.

**Fourth International Symposium on Hyperthermic Oncology**
*2–6 July 1984* *Aarhus, Denmark*
J. Overgaard, Institute of Cancer Research, Radiumstationen, DK-8000 Aarhus C, Denmark.

**IUPHAR Ninth International Congress of Pharmacology**
*29 July–3 August 1984* *Barbican Centre, London, UK*
Congress Secretariat, Conference Associates, 34 Stanford Road, London W8 5PZ, UK.

**Second Terry Fox Cancer Conference: Epigenetic Regulation of Cancer**
*2–4 August 1984* *University of British Columbia, Vancouver, Canada*
The Terry Fox Cancer Conference, University of British Colombia, Dept. of Anatomy, 2177 Wesbrook Mall, Vancouver, B.C., Canada V6T 1W5.

**International Congress on the Endocrinology of the Breast**
*September 1984* *Turin, Italy*
Alberto Angeli, Luigi Dogliotti, Patologia Speciale Medica D., Universita degli Studi di Torino, Via Genova 3, 10126 Torino, Italy.

**Ninth International Subcellular Methodology Forum: Antibody combining sites and their exploitation in cellular studies**
*3–6 September 1984* *Guildford, UK*
Dr. E. Reid, Guildford Academic Associates, 72 The Chase, Guildford GU2 5UL, UK.

**Fifteenth Meeting of the European Tumour Virus Group**
*19–24 September 1984* *Urbino, Italy*
Christina d'Addazio, Gen. Sec., XV Meeting ETUG, Dept. of Virology, Istituto Superiore di Sanita, 299 Viale Regina Elena, 00161 Roma, Italy.